# Ballistic Limit of UHMWPE Composite Armor under Impact of Ogive-Nose Projectile

**DOI:** 10.3390/polym14224866

**Published:** 2022-11-11

**Authors:** Li Ding, Xiaohui Gu, Peihui Shen, Xiangsheng Kong

**Affiliations:** 1 School of Mechanical Engineering, Nanjing University of Science and Technology, Nanjing 210094, China; 2 Nanjing Changjiang Electronics Group Co., Ltd., Nanjing 210037, China

**Keywords:** ballistic limit, composite armors, ogive–nose projectile, theoretical analysis, ultra–high molecular weight polyethylene

## Abstract

The ballistic response of armor has been widely used to evaluate its feasibility and advantages as a protective structure. To obtain the ballistic performance and ballistic limitations of composite armor, a type of ultra–high molecular weight polyethylene (UHMWPE) composite armor is designed, which is composed of UHMWPE laminates and steel face sheets of Q235. The total thickness of the armor is 53 mm, with an in–plane dimension of 300 mm × 300 mm. Then, an experimental study of the ballistic impact response of composite armor subject to a typical ogive–nose projectile was carried out. In the velocity range of 501.1 to 1026.1 m/s, the 14.5 mm caliber armor–piercing projectile could penetrate through the composite armor. At the velocity of 433.3 m/s, the A–P projectile was embedded in the armor, leaving a bulge mark on the back sheet. Therefore, 467.2 m/s is taken as the ballistic limit of the armor under the impact of the ogive–nose projectile. In addition, a corresponding numerical simulation model is also established to predict the ballistic limit of the projectile. The numerical predictions are consistent with the experimental results. The ballistic limit obtained from the numerical simulation results is 500 m/s, which is acceptable with a relative error of 7.02%. The failure mechanism of the composite armor is also obtained. Petaling is the main dominant failure mode for both face sheets, while delamination and shear failure dominate the penetration process of UHMWPE laminates. Finally, the perforation mechanism of composite armor under the impact of an A–P projectile is analyzed with theoretical models to predict the residual velocity, the work performed during the perforation, and the resisting stress of *σ*_s_ in the cavity–expansion model. The experimental and numerical simulation results can provide necessary data in the analysis of the composite structure’s dynamic response under the impact of sharp head penetrators. The research results present the ballistic performance, failure mechanism, and ballistic limit of the composite armor under the impact of a typical ogive–nose projectile, which can be significant in the design of composite armor in the areas of ship shield, fortifications protection, and bulletproof structures against threats from sharp head penetrators.

## 1. Introduction

Multilayered armor systems have been widely used in numerous ballistic and bulletproof applications, including bulletproof helmets, vests, and other armor parts, providing an acceptable range of protection for soldiers and structures [1,2,3,4,5,6]. Due to the advantages of high stiffness and low density, composites reinforced with ultra–high molecular weight polyethylene (UHMWPE) fibers are increasingly being used in the field of national defense as parts of lightweight armor systems to protect fortifications and structures from ballistic impacts [7,8,9]. The ballistic performance of monolithic UHMWPE composite under the impact of a blunt projectile has been studied [10,11,12,13,14], and the failure mechanisms analyzed. A common design of composite armor is a sandwich structure, which is made of thin face sheets and low–density non–metal cores. In addition, the ballistic impact response of a sandwich structure consisting of UHMWPE under a blunt projectile has also been investigated [15,16,17,18,19]. Deflection and bulging [4] consisting of shear plugging, formation of a transition plane, and bulging are the failure modes of UHMWPE [11], which allow it to have an excellent ability to resist the penetration of blunt projectiles.

It is reported that the bulletproof properties of composite materials are greatly discounted under the ballistic impact of projectiles with a sharp head [17,20,21]. However, little is reported on the ballistic performance of the UHMWPE or composite armors containing UHMWPE laminates under the impact of sharp head projectiles. Whether the ballistic performance of the composite armor can be predicted using numerical simulation has not been discussed yet. In addition, to evaluate the resistance of composite armors, especially with the sandwich structure containing UHMWPE laminates, the failure mode and failure mechanism of UHMWPE should also be further studied. An experimental study is one of the basic methods of scientific research, which provides the most direct evidence to help understand physical phenomena. However, the penetration process of the ballistic impact can hardly be observed in experiments. Therefore, due to the limits of testing conditions and the costs of the investment, it is difficult to carry out the relevant research that depends on experiments. Numerical simulations are similar to experimental research that is based on theoretical research [10,14,22]; they are efficient but irrelevant in external conditions. With the help of computer realization, the laws of penetration and dynamic response of the target can be obtained with accurate simulation models. The intermediate process in the penetration process could also be observed, and the crucial parameters could be extracted in the post–processing section to help understand the mechanism of the physical experiment better.

In this paper, a sandwich structure of UHMWPE composite armor is designed, which is made of two pieces of UHMWPE laminates in the middle and Q235 steel face sheets. The ballistic performance of the composite armor is studied systematically both from experiment and numerical simulation. The ballistic limit of UHMWPE composite armor under the impact of an ogive–nose penetrator is obtained, and the failure mechanism of the armor is analyzed. Finally, typical theoretical models are used to predict the residual velocity, the work performed during the perforation, and the resisting stress of *σ*_s_ in the cavity–expansion model to help better understand the penetration process of the composite armor under the impact of sharp head projectiles.

## 2. Configuration of the Armor and Projectile

### 2.1. Design of the Armor

**Figure 1:**
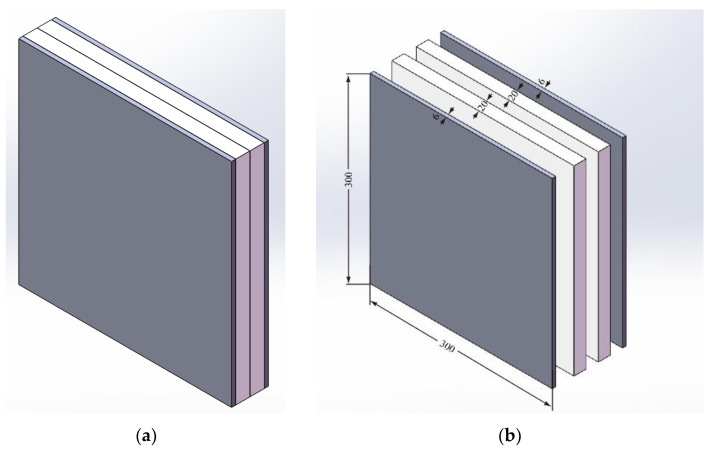
Structures of UHMWPE composite armor. (**a**) Assembly drawing. (**b**) Component drawing.

As shown in Figure 1, the UHMWPE composite armor is made up of two pieces of UHMWPE laminates in the middle and Q235 steel face sheets. Typical UHMWPE laminate with a material grade of FDB4-HW-S1 is selected. Each piece of UHMWPE laminate has a thickness of 20 mm, and each Q235 steel face sheet has a thickness of 6 mm. Each layer of armor has the same in–plane dimensions of 300 mm × 300 mm, with a total thickness of 52 mm. The thin binder layer is replaced in each panel, then followed by pressing to obtain the overall panel structure of the composite armor. Due to the existence of a binder layer, the total thickness of the composite armor may increase from 1 mm to 53 mm.

The material properties of Q235 steel are presented in Table 1, which is provided by the manufacturer of Wuhan Iron & Steel Co., Ltd., Wuhan, China.

### 2.2. Structure of the Projectile

**Figure 2:**
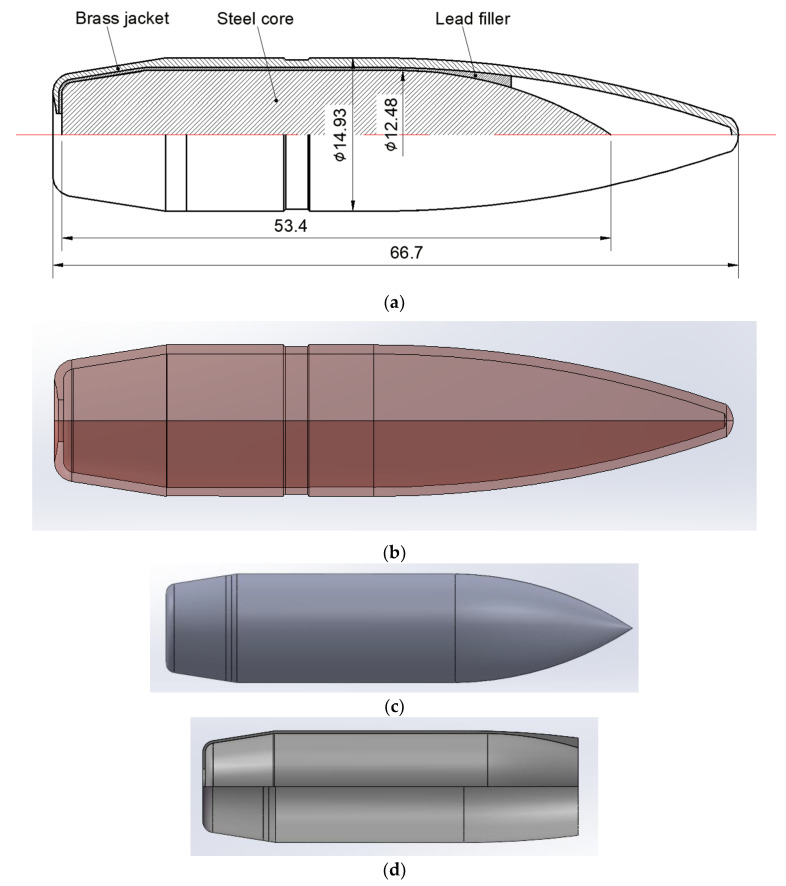
Structure of a 14.5 mm caliber armor–piercing projectile. (**a**) Geometric structure. (**b**) Brass jacket. (**c**) Steel core. (**d**) Lead filler.

The structure of the standard Chinese projectile of 14.5 mm caliber armor–piercing (A–P) projectile is shown in Figure 2. The main geometric parameters of the projectile are presented in Figure 2a, with a total diameter of 14.93 mm and a length of 66.7 mm. The projectile is mainly composed of the brass jacket, steel core, and lead filler. The steel core with an ogive–nose head is made of a non–deformable hardened steel core, which will mainly contribute to the penetration performance of the projectile. The A–P core has a diameter of 12.48 mm and a length of 53.4 mm, with a mass of 40.2 to 41 g. Generally, the jacket is made of gliding brass, and the filler is made of lead, which serves to protect the barrel from the core, enhance the sealing effect and provide an optimized shape for flight in the air. They have a relatively small effect during the penetration, and they are not modeled in the following numerical simulation [23,24,25].

The ballistic limit or limit velocity is the velocity required for a particular projectile to reliably penetrate a particular piece of material. In other words, a given projectile will not pierce a given target when the projectile velocity is lower than the ballistic limit [26]. In addition, it is also important to evaluate the resistance of the armor. Due to the unknown perforation mechanism and the unknown ballistic limit of composite armor under the ballistic impact, especially the sharply pointed projectile, an experiment of the UHMWPE composite armor under the ballistic impact of the A–P projectile was carried out first.

## 3. Experimental Details and Results

### 3.1. Design of the Experiment

**Figure 3:**
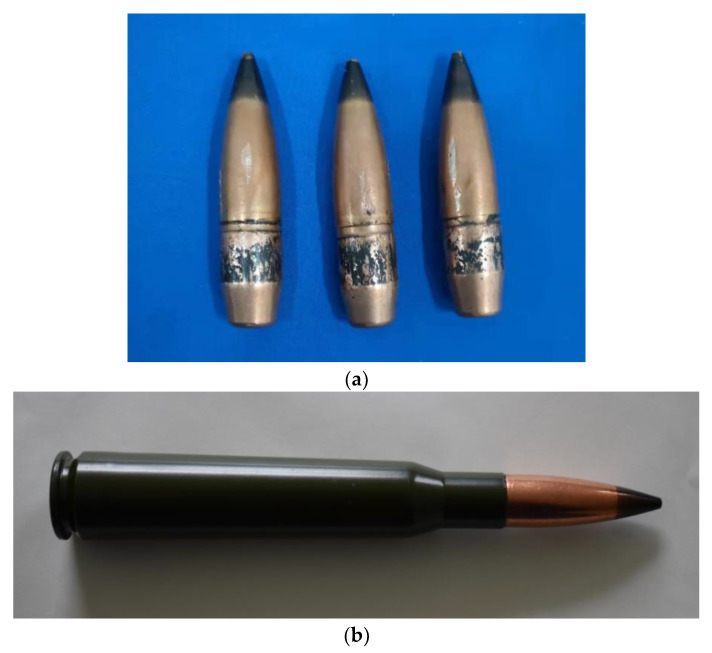
State of the projectile in the experiment. (**a**) 14.5 mm caliber A–P projectile. (**b**) Assembly of the projectile in the cartridge.

Figure 3 shows the 14.5 mm caliber A–P projectile and the state of the assembly of the projectile in the cartridge. By adjusting the quantity of the propellent in the cartridge, the pre–set velocities of the A–P projectile can be acquired.

**Figure 4:**
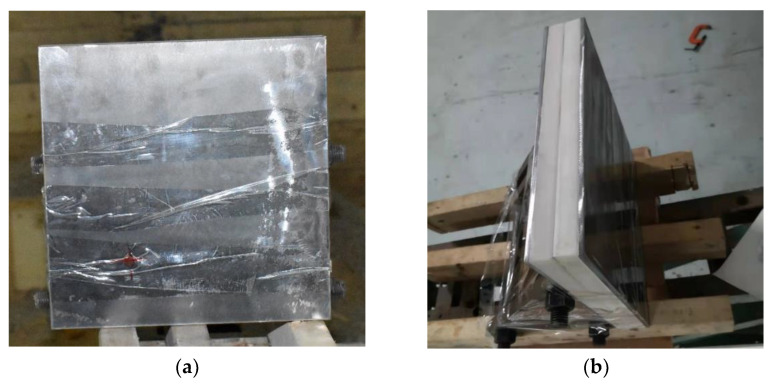
Photograph of the armor in the experiment. (**a**) front view. (**b**) side view.

**Figure 5:**
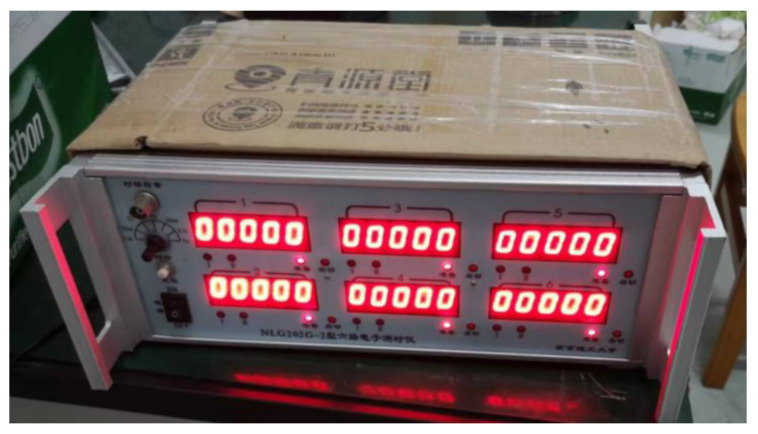
Photograph of the electronic time–measuring instrument.

**Figure 6:**
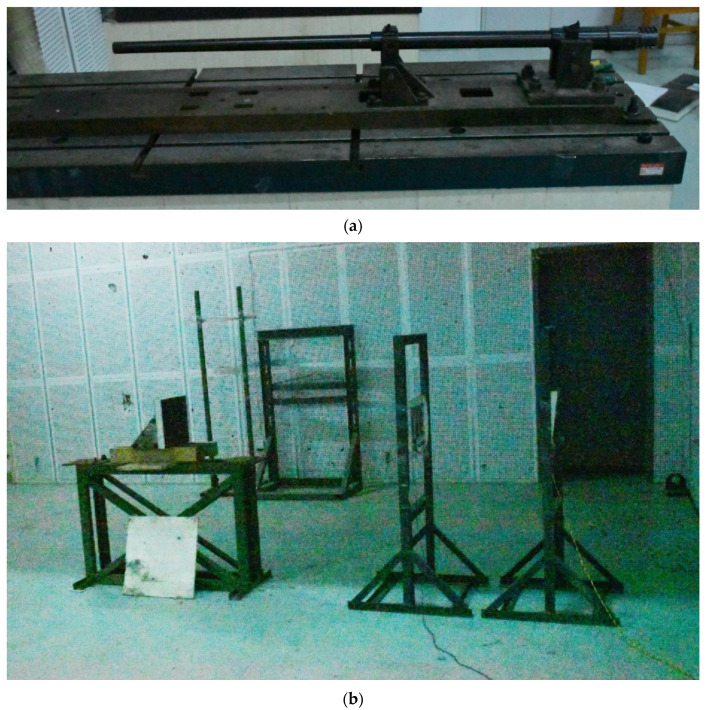
Configuration of the ballistic impact experiment. (**a**) 14.5 mm caliber smooth–bore gun. (**b**) Layout of the targets.

Figure 4 shows the states of the composite armors used in the experiment. The armors were clamped to the rear base on the steel shelf. The rear base was a triangle–shaped bracket, which was fixed to the steel shelf with bundles of iron wire. Figure 5 presents the electronic time–measuring instrument with six channels to capture the signal when the penetrator perforates through the tinfoil target. The electronic time–measuring instrument is of high sensitivity and is able to record the minimum time difference of 1 μs. The layout of the ballistic impact experiment is presented in Figure 6. The 14.5 mm caliber smooth–bore gun was mounted on a rigid platform. The distance between the gun muzzle and armor is about 4 m. To measure the impact velocity of the projectile, two tinfoil targets were placed in front of the armor. The composite armor was placed on a steel shelf at the same height as the ballistic gun.

### 3.2. Experimental Results

The perforation results of composite armor by the A–P projectile are listed in Table 2. For the unknown perforation mechanism and the unknown ballistic limit of composite armor under the ballistic impact of the sharply pointed projectile especially, the impact velocities *v*_i_ of the ogive–nose projectile were set at 1026 m/s initially to much lower velocities. In the velocity range of 501.1 to 1026.1 m/s, the A–P projectile could penetrate through the composite armor. While at the velocity of 433.3 m/s, the A–P projectile was embedded in the armor, leaving a bulge mark on the back sheet. The diameter of the entrance hole on the front sheet was about 14–16 mm, while the diameter of the outlet on the back sheet was about 13–24 mm. The entrance dimension discrepancies are small on the front sheet, and the divergence increased with the increase in impact velocity.

**Figure 7:**
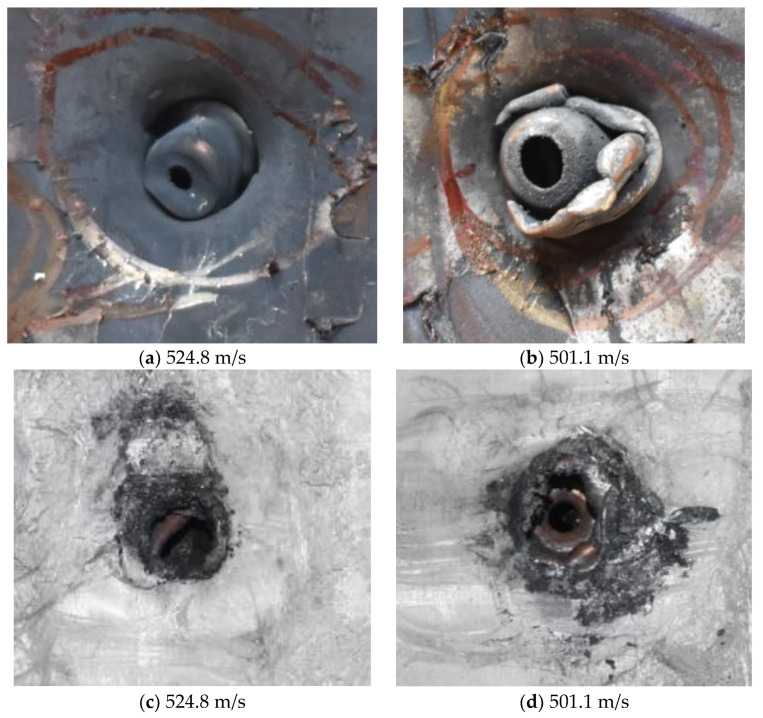
The striped brass jacket and lead filler in the perforation of the front sheet at different impact velocities ((**a**,**b**) for the front view, (**c**,**d**) for the back view).

Figure 7 shows the state of a stripped brass jacket and lead filler. Figure 7a,c shows the front and back views of the perforation on the front sheet at the velocity of 524.8 m/s, and Figure 7b,d shows the front and back views of the perforation at the velocity of 501.1 m/s. The ablative phenomenon can be observed, especially on the back view of the front sheet, resulting from a severe interaction during the penetration.

**Figure 8:**
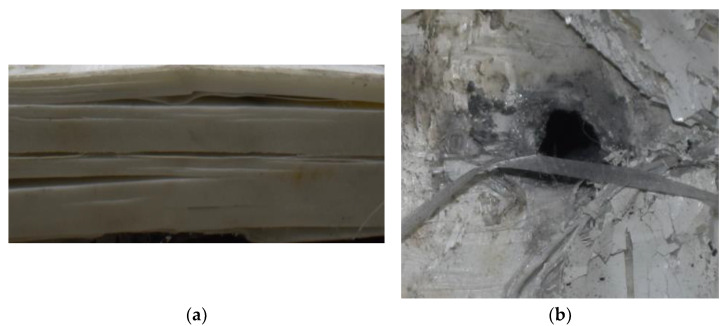
Failure states of the PE laminates. (**a**) Side view of PE laminates. (**b**) Outlet of PE laminate.

Figure 8 shows the failure states of the PE laminates. As shown in Figure 8a, in the side view of the PE laminates, the obvious phenomenon of delamination can be observed. As presented in Figure 8b, a penetration cavity was formed by the ogive–nose penetrator with relatively neat cutting edges accompanied by a charring layer. In addition, the PE laminate exhibited an extent of fibrillation, and the bare bunches of fibers can be observed around the penetration hole.

**Figure 9:**
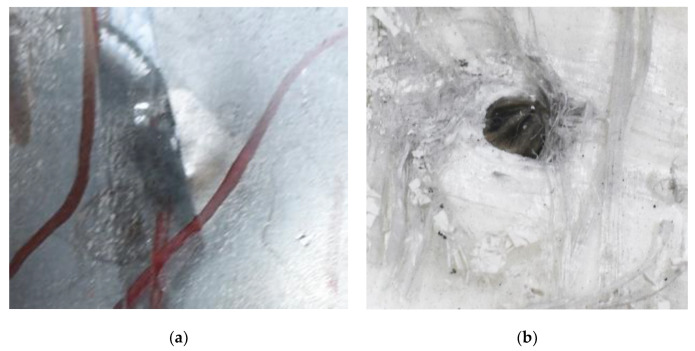
Additional details for the perforation results at the impact velocity of 433.3 m/s. (**a**) Indentation of back plate by penetration. (**b**) Embedded penetrator.

Figure 9 presents additional detail on the perforation results at the impact velocity of 433.3 m/s, with the penetrator embedded in the armor. An obvious indentation was formed on the back of the back sheet, as shown in Figure 9a. After the back sheet was removed, the head of the steel core of the A–P projectile can be seen in Figure 9b, accompanied by an extent of fibrillation.

**Figure 10:**
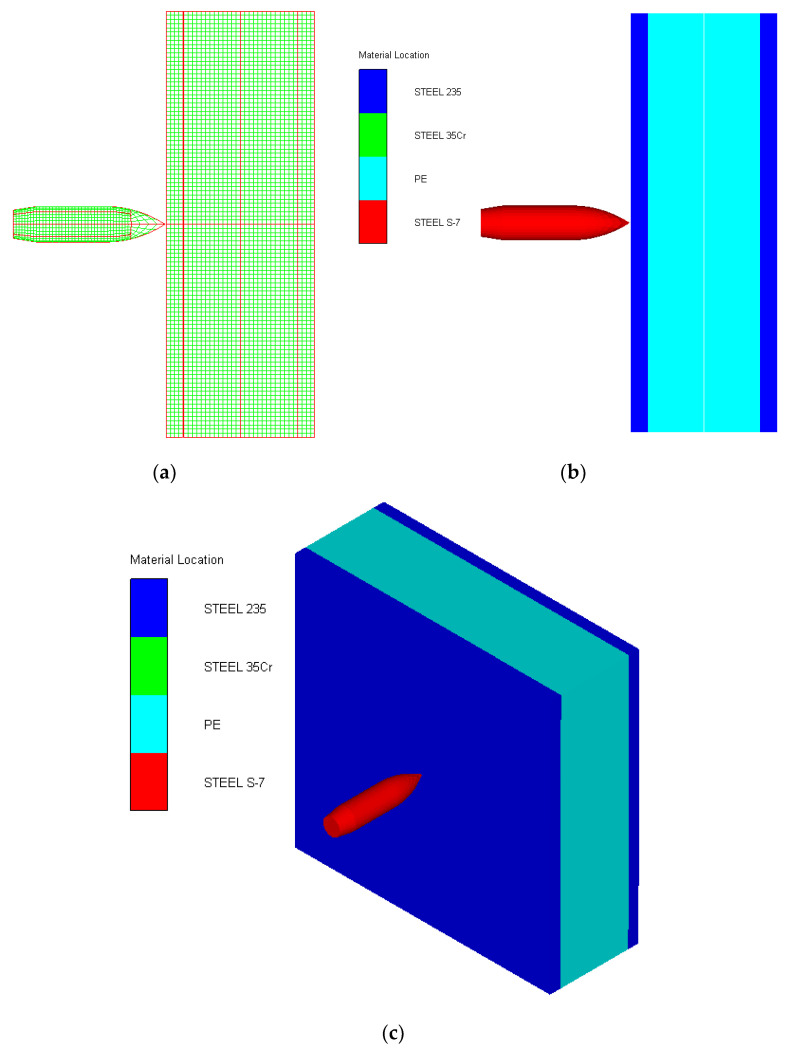
Numerical model of UHMWPE composite armor and the sharp head penetrator. (**a**) Grid model. (**b**) Side view of numerical model. (**c**) Isometric side view of numerical model.

Regarding the results of Table 2 and Figure 7, Figure 8 and Figure 9, it can be summarized from the perforation results that: (1) The average velocities of 501.1 m/s and 433.3 m/s can be taken as the ballistic limits of the UHMWPE composite armor under the impact of the ogive–nose penetrator at 467.2 m/s. (2) Petaling, as the main dominant failure mode for both face sheets, can be observed within the range of impact velocity of 501 m/s to 1026 m/s. The surface of both face sheets stays relatively flat, with small overall deformation except for the protruding petal–shaped holes. Small pieces of petals accompanied by gaped rifts formed the perforation. (3) Delamination and shear failure dominate the penetration process of UHMWPE laminates. Due to the low interlaminar stiffness and strength in the PE laminate, delamination is prevalent through the panel’s thickness, as can be seen in Figure 10a. (4) The charring layer on the front steel plate can be observed, and more severe ablation could be noticed at the impact velocity of around 1000 m/s.

## 4. Numerical Simulation and Analysis

### 4.1. Setup of Numerical Model

To predict the dynamic response and obtain the ballistic limit of UHMWPE composite armor under the ballistic impact of the A–P core, three–dimensional numerical models are carried out using the AUTODYN nonlinear software. The version of AUTODYN is v11.0 in the software of ANSYS 11.0, located in Nanjing, China.

As shown in Figure 10, the 3D Lagrange algorithm is adopted for all of the components in numerical simulation. The half 3D model is carried out with a mesh size of about 1.2 mm per grid. A hexahedral structured grid is used to model both the projectile and the composite armor. The numerical simulation model is composed of about 810 thousand nodes and 800 elements. On the edge of the target, fixed boundaries are used to constrain the movement of the armor. The boundary conditions are applied on the edges of both the face and back sheets. Different initial velocities are applied to the ogive–nose head penetrator to simulate the dynamic penetration behavior with different impact velocities. The material models and the parameters will be described below.

As presented in Table 3, the material models for the penetrator, face sheet, and UHMWPE laminate are listed. For steel, the shock equation of state, also called Grüneisen, is employed in conjunction with the Johnson–Cook constitutive model to simulate the dynamic response under ballistic impact. The Grüneisen EOS [27] can be used to describe how the materials interact with the shock wave and are based on Hugoniot’s relation between the v_s_. and the *v*_p_, as *v*_s_ = *c*_0_ + *sv*_p_, where v_s_. is the shock wave velocity, *v*_p_ is the material particle velocity, *c*_0_ is the wave speed, and *s* is a material–related coefficient. The expression of the equation of state of Grüneisen for the compressed state is:

(1) $$p=\frac{\rho_{0}C^{2}\mu \left[1+\left(1-\frac{\gamma_{0}}{2}\right)\mu -\frac{a}{2}\mu^{2}\right]}{\left[1-\left(S_{1}-1\right)\mu -S_{2}\frac{\mu^{2}}{\mu +1}-S_{3}\frac{\mu^{3}}{{\left(\mu +1\right)}^{2}}\right]}+\left(\gamma_{0}+a\mu \right)E.$$

In the expanded state,

(2) $$p=\rho_{0}C^{2}\mu +\left(\gamma_{0}+a\mu \right)E$$

where *C* is the intercept of the velocity curve between the shock wave and particle; *S*_1_, *S*_2_, and *S*_3_ represent the slope of the *v*_s_ − *v*_p_ curve; *γ*_0_ is the coefficient of the Grüneisen; *a* is the one–order correction of *γ*_0_. *μ* = *ρ*/*ρ*_0_ − 1 is a non–dimensional coefficient based on initial and instantaneous material densities. The parameters of the Grüneisen equation of state are listed in Table 4.

The Johnson–Cook model [28,29] incorporates the effect of strain rate–dependent work hardening and thermal softening, which is given by:

(3) $$\sigma =\left(A+B\varepsilon^{n}\right)\left(1+C\ln\frac{\dot{\varepsilon }}{{\dot{\varepsilon }}_{0}}\right)\left(1-T^{*m}\right)$$

where *ε* is the plastic strain, and the temperature factor is expressed as:

(4) $$T^{*}=\frac{T-T_{r}}{T_{m}-T_{r}}$$

where *T*_r_ is the room temperature, and *T*_m_ is the melt temperature of the material. *A*, *B*, *n*, *C*, and *m* are material–related parameters. The material parameters of S-7 tool steel and Q235 steel are presented in Table 5.

The orthotropic material model proposed by Long H. Nguyen et al. [14] was used for modeling the dynamic behavior of the UHMWPE layer subjected to ballistic impact. The material models consist of a nonlinear equation of the state of orthotropic, a strength model, and a failure model. The constitutive response of the material in the elastic regime is described as the orthotropic EOS composed of volumetric and deviatoric components. The pressure is defined by:

(5) $$\begin{array}{l} P=P\left(\varepsilon_{vol},e\right)-\frac{1}{3}\left(C_{11}+C_{21}+C_{31}\right)\varepsilon_{11}^{d}-\frac{1}{3}\left(C_{12}+C_{22}+C_{32}\right)\varepsilon_{22}^{d}\\ -\frac{1}{3}\left(C_{13}+C_{23}+C_{33}\right)\varepsilon_{33}^{d} \end{array}$$

where *C*_ij_ are the coefficients of the stiffness matrix, $\varepsilon_{ij}^{d}$ refers to the deviatoric strains in the principal directions, and the volumetric component $P\left(\varepsilon_{vol},e\right)$ is defined by the Mie–Grüneisen EOS:

(6) $$P\left(\varepsilon_{vol},e\right)=P_{r}\left(v\right)+\frac{\Gamma \left(v\right)}{v}\left[e-e_{r}\left(v\right)\right]$$

where *v*, *e,* and Γ(*v*) represent the volume, internal energy, and the Grüneisen coefficient, respectively. *P_r_*(*v*) is the reference pressure, and *e_r_*(*v*) is the reference internal energy. The quadratic yield surface was adopted as the material strength model to describe the nonlinear, irreversible hardening behavior of the composite laminate:

(7) $$\begin{array}{l} f\left(\sigma_{ij}\right)=a_{11}\sigma_{11}^{2}+a_{22}\sigma_{22}^{2}+a_{33}\sigma_{33}^{2}+2a_{12}\sigma_{11}\sigma_{22}+2a_{23}\sigma_{22}\sigma_{33}\\ +2a_{13}\sigma_{11}\sigma_{33}+2a_{44}\sigma_{23}^{2}+2a_{55}\sigma_{31}^{2}+2a_{66}\sigma_{12}^{2}=k \end{array}$$

where *a*_ij_ are the plasticity coefficients, and *σ*_ij_ represent the stresses in the principal directions of the material. In addition, the state variable, *k*, is used to define the border of the yield surface. It is described with a master and stress–effective plastic strain curve defined by ten piecewise points to consider the effect of strain hardening.

In the numerical models, the failure model of the orthotropic material is based on a combined stress criterion given as follows:

(8) $${\left(\frac{\sigma_{ii}}{S_{ii}\left(1-D_{ii}\right)}\right)}^{2}+{\left(\frac{\sigma_{ij}}{S_{ij}\left(1-D_{ij}\right)}\right)}^{2}+{\left(\frac{\sigma_{ki}}{S_{ki}\left(1-D_{ki}\right)}\right)}^{2}\geq 1\;\mathrm{for}\;i,j,k=1,2,3$$

where *S* is the failure strength in the respective directions of the material, and *D* is the damage parameter following a linear relationship with stress and strain, as shown below:

(9) $$D_{ii}=\frac{L\sigma_{ii,f}\varepsilon_{cr}}{2G_{ii,f}}$$

where *L* is the characteristic cell length, *ε*_cr_ refers to the crack strain, and *G*_ii,f_ presents the fracture energy in the direction of damage.

The constants for the orthotropic equation of state are presented in Table 6, and the parameters for orthotropic yield strength are shown in Table 7.

### 4.2. Numerical Results and Analysis

Table 8 presents the numerical simulation results of the A–P core penetrating the composite armor. *v*_i_ and *v*_r_ are the impacts and residual velocities of the ogive–nose penetrator. *p* is the depth of penetration. Due to the experimental results, the impact velocity is set from 430 m/s to 700 m/s. With the increased impact velocity, the penetration depth gradually increased. When the impact velocity reached 500 m/s, the ogive–nose penetrator could just perforate the composite armor.

**Figure 11:**
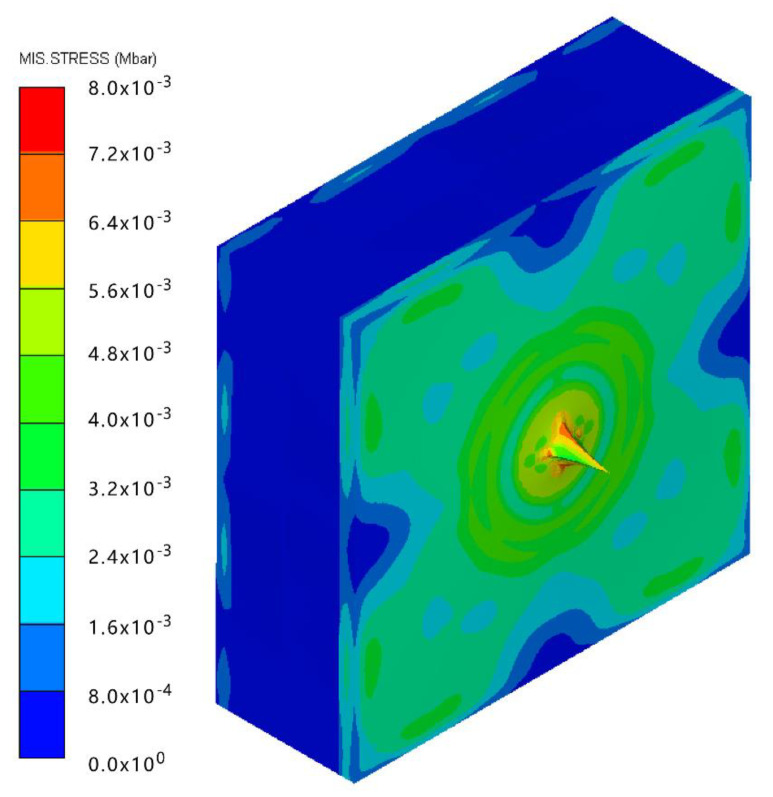
The contour of Von–Mises stress at the impact velocity of 500 m/s.

The contour of Von–Mises stress at the impact velocity of 500 m/s is shown in Figure 11. It can be inferred that the maximum stress exceeds the yield stress of the steel plate, and Q235 back plate is pierced. Therefore, the velocity of 500 m/s can be considered as the ballistic limit of the composite armor, which is much higher than the 467.2 m/s obtained from the experimental results. The numerical simulation results are acceptable, with a relative error of 7.02%.

### 4.3. Perforation Models and Analysis

(1) Principle of energy conservation

The energy balance for the perforation is given by

(10) $$\frac{1}{2}mv_{i}^{2}=\frac{1}{2}mv_{r}^{2}+W$$

(11) $$W=W_{Q235}+W_{\mathrm{PE}}$$

where *m* is the mass of the projectile, *v*_i_ is the impact velocity, *v*_r_ is the residual velocity, and *W* is the work performed during perforation. The mass of the A–P core was set at 40.4 g, then the work conducted during the perforation of the composite armor could be calculated, as listed in Table 9. The value of *W* stayed stable from 5.05 kJ to 5.09 kJ, which means that dissipated energy in the petaling stays stable at around 5 kJ. At the ballistic limit from the numerical results, 500 m/s, the dissipated energy is the same as the work performed at a higher velocity after perforation. So, the principle of energy conservation can be applied here.

(2) Lambert–Jonas model

The Lambert–Jonas model [26,30,31,32] can provide a reasonable fit to predict the residual velocity of the penetrator after perforation. The model can be expressed as

(12) $$v_{r}=\{\begin{array}{c} 0,0\leq v_{i}\leq v_{\mathrm{bl}}\\ \alpha {\left(v_{i}^{p}-v_{\mathrm{bl}}^{p}\right)}^{1/p},\;v_{i}\geq v_{\mathrm{bl}} \end{array}$$

where *v*_i_, *v*_r,_ and *v*_bl_ are the impact, residual, and ballistic limit velocity in normal impact. *α* and *p* are the coefficients, where 0 ≤ *α* ≤ 1 and *p* > 1. Based on the numerical simulation results, the Lambert–Jonas model can be established to predict the residual velocity of the A–P core after perforating the PE composite armor.

**Figure 12:**
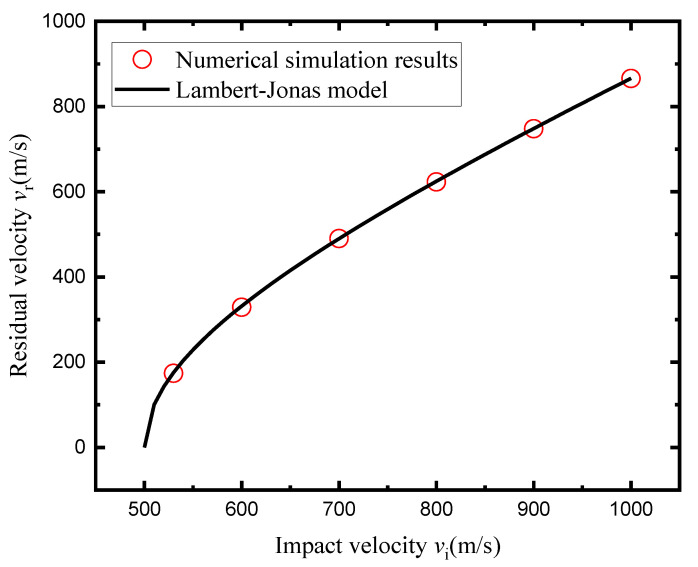
Comparison between the Lambert–Jonas model and the numerical simulations.

When the model with *p* = 2, the coefficient *α* can be set as 1, and the model can be justified based on the energy conservation law [33]. This model can be written as

(13) $$v_{r}=\{\begin{array}{c} 0,0\leq v_{i}\leq v_{\mathrm{bl}}\\ {\left(v_{i}^{2}-v_{\mathrm{bl}}^{2}\right)}^{1/2},\;v_{i}\geq v_{\mathrm{bl}} \end{array}$$

the predicted *v*_r_ − *v*_s_ curve and the simulation results are presented below. As shown in Figure 12, the Lambert–Jonas model can be an effective method in predicting the residual velocity of the A–P core after perforation. In addition, the perforation process can be regarded as a rigid body penetration.

(3) Cavity–Expansion Model

**Figure 13:**
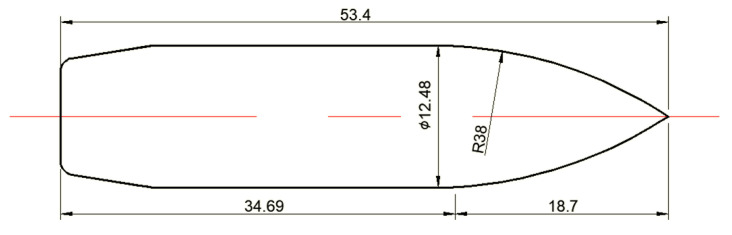
Geometry and dimensions (in mm).

As the A–P core has a diameter of 12.48 mm and a length of 53.4 mm, the composite armor with a thickness of 53 mm can be considered an intermediate target. The square armor has a width of 300 mm, which is about 24 times the diameter of the A–P core. Thus, the cylindrical cavity expansion can be used to predict the ballistic limit of the A–P core. Figure 13 shows the dimensions of the A–P core. The caliber–radius–head (CRH) is 3.05, which is also denoted as *ψ*.

Coefficient *k*_1_ is expressed as

(14) $$k_{1}=\left(4\psi^{2}-4\psi /3+1/3\right)-\frac{4\psi^{2}\left(2\psi -1\right)}{\sqrt{4\psi -1}}\sin^{-1}\left[\frac{\sqrt{4\psi -1}}{2\psi }\right]$$

The radial stress *σ*_r_ at the cavity surface versus cavity expansion velocity *V* is given by [34]

(15) $$\sigma_{r}=\sigma_{s}+\rho_{t}BV^{2}$$

where *σ*_s_ is the quasi–static radial stress required to open the cylindrical cavity, *ρ*_t_ is the density of the target, and *B* is a dimensionless constant. *σ*_s_, *b*, and *B* are obtained from [23]

(16) $$\sigma_{s}=\frac{Y}{\sqrt{3}}\left\{1+{\left[\frac{E}{\sqrt{3}Y}\right]}^{n}\int_{0}^{b}\frac{{\left(-\ln x\right)}^{n}}{1-x}dx\right\}$$

*b* = 1 − γ^2^ (17)

(18) $$B=\frac{1}{2}\left\{\frac{1}{(1-\nu )\sqrt{1-\alpha^{2}}}\ln\left[\frac{1+\sqrt{1-\alpha^{2}}}{\alpha }\right]+\gamma^{2}-2\ln\left[\gamma \right]-1\right\}\;$$

where *Y* is, the yield stress and *ν* is Poisson’s ratio of the target. *α* and *γ* are given by

(19) $$\alpha^{2}=\frac{\sqrt{3}(1-2\nu )}{2(1-\nu )}\left(\frac{\rho_{t}V^{2}}{Y}\right)$$

(20) $$\gamma^{2}=\frac{2(1+\nu )Y}{\sqrt{3}E}$$

Furthermore, a rigid ogive–nosed projectile, with the impact velocity of *v*_i_, the ballistic limit of *v*_bl_ and the residual velocity *v*_r_, is given by

(21) $$v_{bl}={\left(\frac{2\sigma_{s}}{\rho_{p}}\frac{h}{\left(L+k_{1}l\right)}\right)}^{1/2}{\left[1+C+\frac{2}{3}C^{2}\right]}^{1/2}$$

(22) $$v_{r}=v_{bl}{\left[{\left(\frac{v_{i}}{v_{bl}}\right)}^{2}-1\right]}^{1/2}{\left[1-C+\frac{1}{2}C^{2}\right]}^{1/2}$$

where *C* is a small parameter related to the target inertia. When target inertia is neglected, the ballistic limit of *v*_bl_ and the residual velocity *v*_r_ can be simplified as [23,25,35] as

(23) $$v_{bl}={\left(\frac{2\sigma_{s}}{\rho_{p}}\frac{h}{\left(L+k_{1}l\right)}\right)}^{1/2}$$

(24) $$v_{r}=v_{bl}{\left[{\left(\frac{v_{i}}{v_{bl}}\right)}^{2}-1\right]}^{1/2}$$

where the residual velocity *v*_r_ is the same as the Lambert–Jones model in Equation (13).

Based on the constitutive models of the target materials, the quasi–static radial stress *σ*_s_ can be expressed as [36]

(25) $$\sigma_{s}=\frac{Y}{\sqrt{3}}\left[1+\ln\left(\frac{E}{\sqrt{3}Y}\right)\right]+\frac{\pi^{2}H}{18}$$

where *E* and *H* are Young’s modulus and the constant tangent modulus in the plastic region if the stress versus strain curve of the target can be expressed as

(26) $$\sigma =\{\begin{array}{c} E\varepsilon \;,\;\sigma <Y\\ Y+H\varepsilon ,\;\sigma \geq Y \end{array}$$

Thus, the value of *σ*_s_ for the Q235 face sheets can be calculated. For UHMWPE laminates, there may not be a mature model to predict the quasi–static radial stress required to open the cylindrical cavity, but the range of the *σ*_s_ can be estimated from the empirical formula [37,38] below,

(27) $$\sigma_{s}=\left(1.33\sim 2\right)Y_{t}$$

When the coefficient is set as the minimum value of 1.33, the value at a relatively low level can be obtained, as listed in Table 10.

For the composite armor composed of Q235 face sheets and UHMWPE laminates, the effective value of *σ*_s_ can range from 2.76 GPa to 4.26 GPa. When the value of effective *σ*_s_ is set as 3.08 GPa, the ballistic limit of the composite armor calculated from Equation (23) is 467 m/s, which is consistent with the value obtained from the numerical simulation results.

In conclusion, the principle of energy conservation and the Lambert–Jonas model can be applied to calculate the work performed during the perforation and the residual velocities of the A–P core after perforation. In addition, the quasi–static radial stress *σ*_s_ required to open the cylindrical cavity can be estimated from the cavity–expansion model. With the value of 3.08 GPa, the predicted ballistic limit is consistent with the numerical simulation results.

## 5. Conclusions

A UHMWPE composite armor made up of two pieces of UHMWPE laminates in the middle and Q235 steel face sheets is proposed, and a study of the ballistic limit of the composite armor under the impact of a typical ogive–nose penetrator was carried out. (1) According to the experimental results, the average velocity of 501.1 m/s and 433.3 m/s can be taken as the ballistic limit of UHMWPE composite armor under the impact of the ogive–nose projectile, which is 467.2 m/s. In comparison, the ballistic limit obtained from the numerical simulation results was 500 m/s, which is acceptable with a relative error of 7.02%. (2) Petaling, as the main dominant failure mode for both face sheets, could be observed within the impact velocity range of 501 m/s to 1026 m/s. Delamination and shear failure dominated the penetration process of UHMWPE laminates. In addition, the charring layer on the front steel plate could be observed, and more severe ablation could be noticed at the impact velocity of around 1000 m/s. (3) Through theoretical models, the perforation mechanism of composite armor under the impact of A–P cores was analyzed. The principle of energy conservation and the Lambert–Jonas model was applied to calculate the work performed during the perforation and the residual velocities. In addition, the quasi–static radial stress *σ*_s_ required to open the cylindrical cavity were estimated from the cavity–expansion model. With the value of 3.08 GPa, the predicted ballistic limit was consistent with the numerical simulation results.

The ballistic limit of the UHMWPE composite armor under the impact of the ogive–nose projectile was considered to be 467.2 m/s, which indicates that the composite armor may not have a strong ability to resist the penetration of sharp head penetrators. In order to enhance the resistance against bullets such as A–P projectiles, UHMWPE should be strengthened, and the structure should be further optimized in future studies.

## Figures and Tables

**Table 1:** Material properties of Q235.

Steel	Yield Strength (MPa)	Tensile Strength (MPa)	Elongation after Break (%)	Poisson’s Ratio (%)	Impact Energy Aku (J)
Q235	305	426	30	0.33	≥27

**Table 2:** Perforation results in the experiment.

*v*_i_ (m/s)	Perforation State in the Front and Back	
1026.1	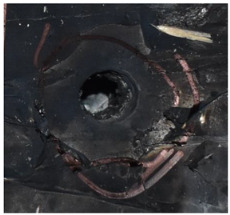	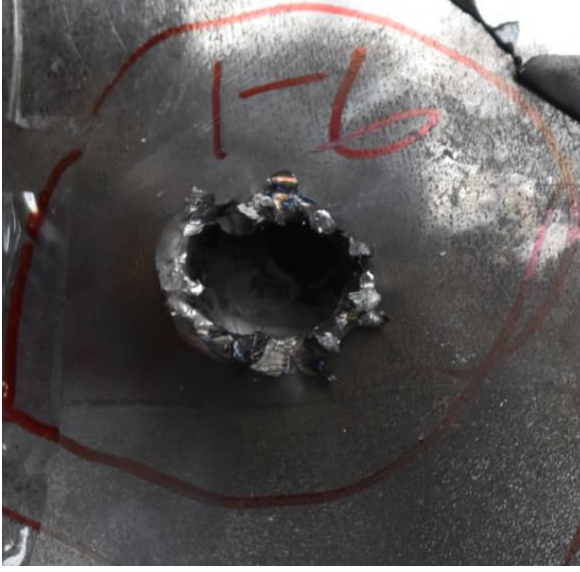
966.6	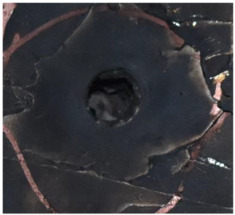	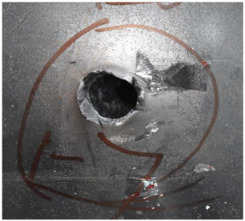
768.7	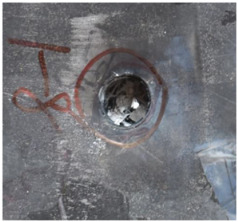	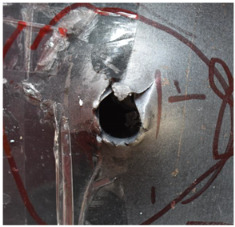
688.4	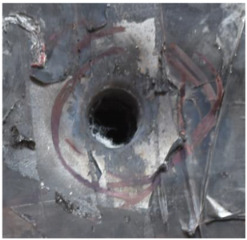	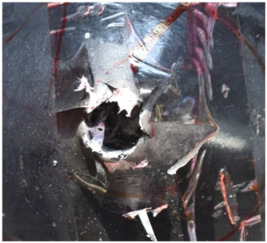
616.8	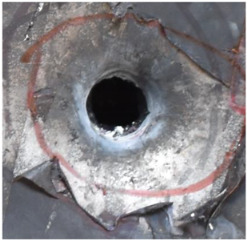	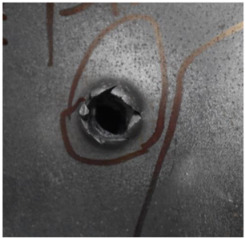
595.4	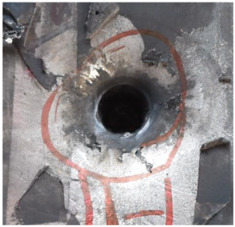	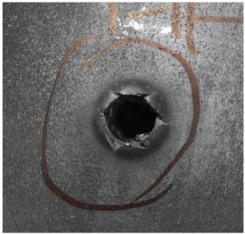
524.8	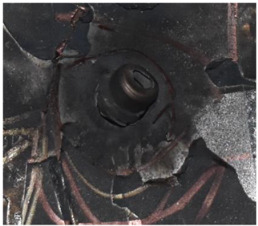	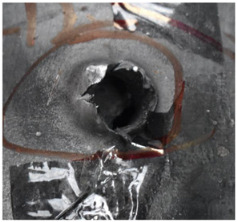
501.1	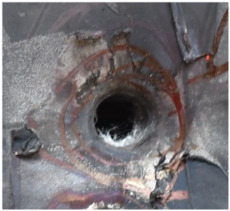	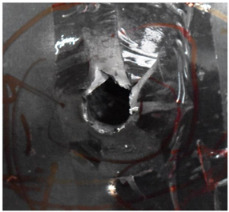
433.3	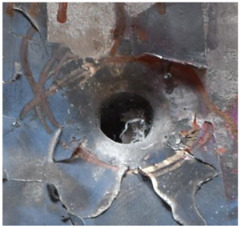	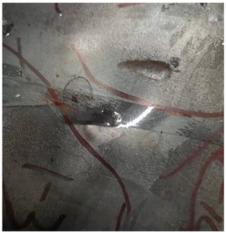

**Table 3:** Material models used in numerical simulation.

Components	Material	*ρ* (g/cm^3^)	Equation of State	Constitutive Model
Penetrator	Steel S-7	7.83	Shock	Johonson-Cook
Face sheet	Steel Q235	7.896	Shock	Johonson-Cook
PE laminates	UHMWPE	0.98	Ortho	Orthotropic Yield

**Table 4:** EOS parameters of S-7 and Q235.

Material	Grüneisen Coefficient	*C* (m/s)	*S*_1_	*S*_2_	*a*
S-7	2.17	4569	1.330	0	0.47
Q235	2.17	4569	1.490	0	0.46

**Table 5:** Material constants for S-7 and Q235.

Steel	*ρ* (g/cm^3^)	*A* (MPa)	*B* (MPa)	*n*	*C*	*m*	${\dot{\varepsilon }}_{0}\;(s^{-1})$	*T*_r_ (K)	*T*_m_ (K)
S-7	7.850	1540	477	0.16	0.016	1.0	1	293	1763
Q235	7.896	350	275	0.36	0.022	1.0	1	293	1793

**Table 6:** Material constants for Orthotropic equation of state.

	Value	Units	Parameters	Value	Units
Reference density	0.98	g/cm^3^	Shear modulus 12	2.0 × 10^6^	kPa
Young’s modulus 11	3.62 × 10^6^	kPa	Shear modulus 23	1.92 × 10^5^	kPa
Young’s modulus 22	5.11 × 10^7^	kPa	Shear modulus 31	2.0 × 10^6^	kPa
Young’s modulus 33	5.11 × 10^7^	kPa	Volumetric response: shock Grüneisen coefficient	1.64	-
Poisson’s ratio 12	0.013	-	Parameter C1	3.57 × 10^3^	m/s
Poisson’s ratio 31	0.5	-	Parameter S1	1.3	-
Reference temperature	293	K	Specific heat	1.85 × 10^3^	J/kgK

**Table 7:** Material constants for Orthotropic yield strength.

Parameters	Value	Units	Parameters	Value	Units
Plasticity constant 11	0.016	-	Eff. plastic strain #1	0	-
Plasticity constant 22	6 × 10^−4^	-	Eff. plastic strain #2	0.01	-
Plasticity constant 33	6 × 10^−4^	-	Eff. plastic strain #3	0.1	-
Plasticity constant 12	0	-	Eff. plastic strain #4	0.15	-
Plasticity constant 13	0	-	Eff. plastic strain #5	0.175	-
Plasticity constant 23	0	-	Eff. plastic strain #6	0.19	-
Plasticity constant 44	1	-	Eff. plastic strain #7	0.2	-
Plasticity constant 55	1.7	-	Eff. plastic strain #8	0.205	-
Plasticity constant 66	1.7	-	Eff. plastic strain #9	0.21	-
/	/		Eff. plastic strain #10	0.215	-
Eff. stress #1	1.48 × 10^3^	kPa	Eff. stress #6	6.0 × 10^4^	kPa
Eff. stress #2	7.0 × 10^3^	kPa	Eff. stress #7	8.0 × 10^4^	kPa
Eff. stress #3	2.7 × 10^4^	kPa	Eff. stress #8	9.8 × 10^4^	kPa
Eff. stress #4	4.0 × 10^4^	kPa	Eff. stress #9	2.0 × 10^5^	kPa
Eff. stress #5	5.0 × 10^4^	kPa	Eff. stress #10	1.0 × 10^6^	kPa

**Table 8:** Numerical simulation results of perforation.

*v*_i_ (m/s)	State of Perforation and Deformation	*p* (mm)	*v*_i_ (m/s)	State of Perforation and Deformation	*v*_r_ (m/s)
430	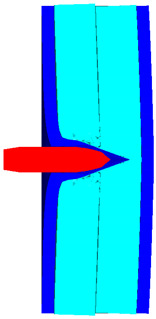	41.55	530	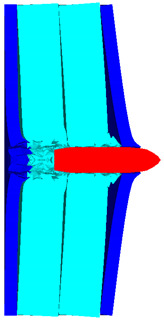	174
450	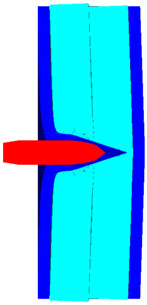	43.15	600	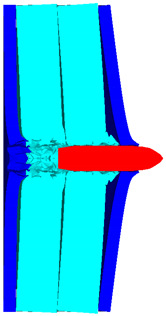	329
500	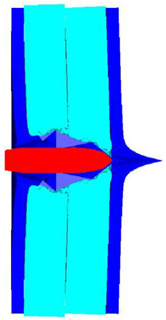	53	700	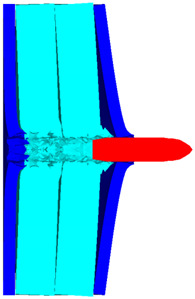	490

**Table 9:** Results of calculated work *W* in the perforation.

*v*_i_ (m/s)	500	530	600	700	800	900	1000
*v*_r_ (m/s)	0	174	329	490	623	748	866
*W* (kJ)	5.05	5.06	5.09	5.05	5.09	5.06	5.05

**Table 10:** The predicted value of quasi–static radial stress *σ*_s._

Materials	*E* (GPa)	Ultimate Tensile/ Compressive Strength (MPa)	*H* (GPa)	*σ*_s_ Predicted from Equation (25) (GPa)	*σ*_s_ Predicted from Equation (27) (GPa)
Q235	200	305	281	2.76	/
UHMWPE	113~124	2700~3200	/	/	3.59~4.26

## Data Availability

The raw and processed data generated during this study will be made available upon reasonable request.
